# NURSING HOME STAFF PERSPECTIVES ON SUPPORTING PERSON-CENTERED CARE IN THE FACE OF RISK TO MAINTAIN QUALITY OF LIFE

**DOI:** 10.1093/geroni/igae098.0078

**Published:** 2024-12-31

**Authors:** Kimberly Curyto, Philip Taylor

**Affiliations:** Chair; Discussant

## Abstract

Nursing home (NH) quality of care efforts emphasize person-centered care as a best practice. Evaluations of person-centered interventions report positive outcomes including improved resident psychological wellbeing, quality of life, and staff job satisfaction. However, prioritizing NH resident personal preferences and values can lead to choices that increase risk, and thus staff must act in ways that balance resident autonomy and safety. Best practices in person-centered care in the NH should honor choice and maintain safety in support of overall resident wellbeing through shared decision making among residents, families and the whole clinical team. The COVID-19 pandemic placed NH residents and staff at risk of decline and death and created barriers to implementing person-centered care. A primary focus of this symposium is to share a range of staff perspectives on supporting NH resident autonomy and safety in the face of risk, including those of NH leaders, mental health providers, and direct care team members such as nursing staff, recreation/activities staff, and social workers. Presentations will highlight the impact of a focus on safety during the increased risk from the pandemic on resident and staff wellbeing, and staff adaptation efforts to ensure person-centered care. Understanding NH staff and leadership perspectives on response to risk while supporting person-centered care will inform future care management during crises and other times of increased concern for safety in NH settings. Important perspectives of paid care workers during the pandemic will be discussed in the context of workforce development efforts.

